# Bioactivity Screening of Antarctic Sponges Reveals Anticancer Activity and Potential Cell Death via Ferroptosis by Mycalols

**DOI:** 10.3390/md19080459

**Published:** 2021-08-14

**Authors:** Gennaro Riccio, Genoveffa Nuzzo, Gianluca Zazo, Daniela Coppola, Giuseppina Senese, Lucia Romano, Maria Costantini, Nadia Ruocco, Marco Bertolino, Angelo Fontana, Adrianna Ianora, Cinzia Verde, Daniela Giordano, Chiara Lauritano

**Affiliations:** 1 Department of Marine Biotechnology, Stazione Zoologica Anton Dohrn, Villa Comunale, 80121 Napoli, Italy; gennaro.riccio@szn.it (G.R.); daniela.coppola@szn.it (D.C.); maria.costantini@szn.it (M.C.); nadia.ruocco@szn.it (N.R.); adrianna.ianora@szn.it (A.I.); c.verde@ibp.cnr.it (C.V.); daniela.giordano@ibbr.cnr.it (D.G.); 2 Istituto di Chimica Biomolecolare, Consiglio Nazionale delle Ricerche, Via Campi Flegrei 34, 80078 Pozzuoli, Italy; nuzzo.genoveffa@icb.cnr.it (G.N.); giusi.senese@icb.cnr.it (G.S.); l.romano@icb.cnr.it (L.R.); angelo.fontana@icb.cnr.it (A.F.); 3 Research Infrastructure for Marine Biological Resources Department, Stazione Zoologica Anton Dohrn, Villa Comunale, 80121 Napoli, Italy; gzazo@inogs.it; 4 Institute of Biosciences and BioResources (IBBR), National Research Council (CNR), Via Pietro Castellino 111, 80131 Napoli, Italy; 5 Dipartimento di Scienze della Terra, dell’Ambiente e della Vita (DISTAV), Università degli Studi di Genova, Corso Europa 26, 16132 Genova, Italy; marco.bertolino@unige.it; 6 Laboratory of Bio-Organic Chemistry and Chemical Biology, Department of Biology, Università di Napoli “Federico II”, Via Cupa Nuova Cinthia 21, 80126 Napoli, Italy

**Keywords:** Antarctica, sponges, drug discovery, mycalols, marine biotechnology

## Abstract

Sponges are known to produce a series of compounds with bioactivities useful for human health. This study was conducted on four sponges collected in the framework of the XXXIV Italian National Antarctic Research Program (PNRA) in November-December 2018, i.e., *Mycale (Oxymycale) acerata*, *Haliclona (Rhizoniera) dancoi*, *Hemimycale topsenti*, and *Hemigellius pilosus*. Sponge extracts were fractioned and tested against hepatocellular carcinoma (HepG2), lung carcinoma (A549), and melanoma cells (A2058), in order to screen for antiproliferative or cytotoxic activity. Two different chemical classes of compounds, belonging to mycalols and suberitenones, were identified in the active fractions. Mycalols were the most active compounds, and their mechanism of action was also investigated at the gene and protein levels in HepG2 cells. Of the differentially expressed genes, ULK1 and GALNT5 were the most down-regulated genes, while MAPK8 was one of the most up-regulated genes. These genes were previously associated with ferroptosis, a programmed cell death triggered by iron-dependent lipid peroxidation, confirmed at the protein level by the down-regulation of GPX4, a key regulator of ferroptosis, and the up-regulation of NCOA4, involved in iron homeostasis. These data suggest, for the first time, that mycalols act by triggering ferroptosis in HepG2 cells.

## 1. Introduction

Marine organisms represent an excellent source of natural products with bioactivities useful for the treatment and prevention of human pathologies, such as cancer, inflammation, and infections. In recent years, the chemistry of natural products derived from marine organisms has received growing interest in the scientific community. In particular, there are fourteen approved pharmaceutical products in clinical use and more than 20 marine natural products in various stages of clinical development (i.e., four compounds in Phase III, twelve in Phase II, and seven in Phase I clinical trials), especially in the field of oncology (https://www.midwestern.edu/departments/marinepharmacology/clinical-pipeline.xml). These compounds have been identified mainly from invertebrates, such as sponges, mollusks, bryozoans, and ascidians. The last two drugs approved in 2020 are Belantamabmafodotin-blmf (Blenrep^TM^) from a mollusk/cyanobacterium for the treatment of relapsed/refractory multiple myeloma and Lurbinectedin (Zepzelca^TM^) from a tunicate for metastatic small cell lung cancer treatment.

It has been known for a long time that sponges may produce interesting compounds with bioactivities useful for human health [1,2,3,4]. In 1907, Richter outlined that the active component of the roasted bath sponge was rich in iodine and was already used by Roger cosmetics against struma [1]. The first marine-derived anticancer compound was cytarabine or Ara-C (Cytosar-U^^®^^), which was developed for clinical use as a synthetic analogue of a C-nucleoside isolated from the Caribbean sponge *Tectitethya crypta* (de Laubenfels, 1949) (ex *Tethya crypta*) [5]. Ara-C was approved in 1969 and is still used to treat acute myelocytic leukemia and non-Hodgkin’s lymphoma [6]. Successively, a synthetic analog of spongouridine, vidarabine or Ara-A (Vira-A^®^), from the sponge *Tectitethya crypta* was approved as an antiviral (1976), while a synthetic analog of halichondrin A, Eribulin (Halaven^®^), from the sponge *Halichondria* (*Halichondria*) *okadai* (Kadota, 1922) was approved for the treatment of drug-refractory breast cancer (2010). Other compounds from sponges in clinical trials are Plocabulin (PM184), actually in Phase II clinical trials and MORAb-202 in Phase I. Both compounds are undergoing trials for the treatment of solid tumors (https://www.midwestern.edu/departments/marinepharmacology/clinical-pipeline.xml). 

The current investigation focuses on antiproliferative bioactivity screening of four sponges collected from two different sites in the framework of the XXXIV Italian National Antarctic Research Program (PNRA) in November–December 2018. Three of them have been previously characterized by morphological analysis of spicules and molecular marker (i.e., 18S, 28S, ITS, and CO1) amplification and were identified as *Mycale (Oxymycale) acerata* Kirkpatrick, 1907, *Haliclona (Rhizoniera) dancoi* (Topsent, 1901), and *Hemigellius pilosus* (Kirkpatrick, 1907) [7].

**Figure 1:**
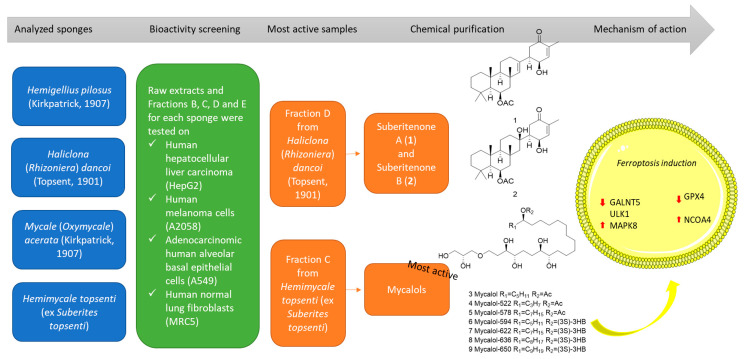
Work pipeline reporting the various experimental steps, the chemical structures of suberitenone A (**1**) and B (**2**) and mycalols, and the main results on the mechanism of action of mycalols, which were identified as the most active compounds in the current study.

Extreme environments, such as the poles, represent an almost untapped source of marine natural products which is still largely unexplored compared to more accessible sites. Studies in extreme environments are rare due to logistic problems and expedition costs. The Southern Ocean represents 9.6% of the world’s oceans and extends approximately 35 million km^2^. The Antarctic region is strongly affected by snow and ice-cover changes, extreme photoperiods, and low temperatures [8]. Due to these harsh characteristics, Antarctic organisms have evolved various physiological and behavioral adaptations [9]. For example, a longer period of larval development has been observed for sponges in Antarctica [10], where they represent the major component of the Antarctic zoobenthos (counting about 400 species; [10,11]). Previous studies have shown that Antarctic sponges may produce bioactive compounds for possible human applications [2,12,13,14,15,16,17,18,19]. This study aims to further explore the Antarctic region and to give an overview of sponge bioactivity from different sites, their chemical composition, and the mechanism of action of the active principles. Herein we report a bioassay-guided fractionation of four Antarctic sponges that led to the identification of two different classes of compounds, suberitenone A (**1**) and B (**2**) and mycalols, as bioactive compounds present in the identified cytotoxic fractions. The mixture containing the marine metabolite mycalol (**3**) and its analogues (**4**–**9**), already reported to possess anticancer activity on anaplastic thyroid carcinoma cells (ATC) [20,21], were identified as the most promising fraction. Thus, the mechanism of action of the mycalol mixture was further investigated at the gene and protein levels (Figure 1).

## 2. Results

### 2.1. Species Identification of Specimen C7

The C7 specimen belongs to the species *Hemimycale topsenti* (Burton, 1929) due to the characteristic areolated surface—presence of a plumose skeleton formed by columns of subtilostyles characterized by oval elongated heads. 

The 663 bp PCR fragment (using primer pair dgLCO1490/dgHCO2198) displayed a 100% of pairwise sequence similarity (85% of query cover) to the species *Hemimycale topsenti* (ex *Suberites topsenti*) mitochondrial partial COI gene voucher NIWA28884 (Accession Number: LN850246.1) (Figure S1). The other three sponges were identified in our previous paper [7] (Table 1).

### 2.2. Chemical Fractionation and Cell Viability on Cancer Cell Lines

Different concentrations (1, 10, and 100 μg mL^−1^) of total MeOH extract (TE) of sampled Antarctic sponges, and their enriched extracts B–E, obtained by solid-phase extraction (SPE) fractionation [22], were screened for their capability to affect the viability of the human cancer cell lines HepG2 (hepatocellular carcinoma), A549 (lung adenocarcinoma) and A2058 (melanoma), and the normal lung cell line MRC5 (Figure S2). 

Bioactivity screening on A2058 cells showed that fraction C, D, and E of the sponge *M. a.* were active only at 100 µg/mL (*p* < 0.1 for fraction C and *p* < 0.001 for fraction D and E), as well as fraction C and D of *H. d.* (*p* < 0.05 for fraction C and *p* < 0.001 for fraction D). For *H. t.*, fraction C was active at both 10 and 100 µg/mL (*p* < 0.001 for both) while total extract and fraction D were active only at 100 µg/mL (*p* < 0.001 for both) (Figure S2). For *H. p.*, total extract, fraction C and fraction E were active only at 100 µg/mL (*p* < 0.01 for total extract and fraction C, while *p* < 0.05 for fraction E; Figure S2).

Regarding A549 cells, *M. a.* fraction C, D, and E were able to significantly reduce A549 cell proliferation only at 100 µg/mL (*p* < 0.05 for fraction C and *p* < 0.001 for fraction D and E,). For *H. d.*, total extract and fraction D were active at 100 μg/mL (*p* < 0.01 for total extract and *p* < 0.01 for fraction D), while fraction C was active both at 10 and 100 μg/mL (*p* < 0.05 and *p* < 0.001, respectively). For *H. t.*, total extract, fraction C and fraction D were active at 100 µg/mL (*p* < 0.001 for all). For *H. p.*, fraction E was the only one active at 100 µg/mL (*p* < 0.001) (Figure S2).

Bioactivity on HepG2 cells showed that fraction D and E of *M*. *a*. were active at 100 µg/mL (*p* < 0.01 and *p* < 0.001, respectively). Fraction C of *H*. *d*. was active at both 10 and 100 µg/mL (*p* < 0.001 for both), total extract and fraction D were active only at 100 μg/mL (*p* < 0.001 for both). For *H*. *t*., total extract, fraction C, D and E were active only at 100μg/mL (*p* < 0.001 for all). *H*. *p*. total extract and fraction D were active at 100 μg/mL (*p* < 0.001 and *p* < 0.01, respectively).

### 2.3. Identification of Compounds in Most Active Fractions

**Figure 2:**
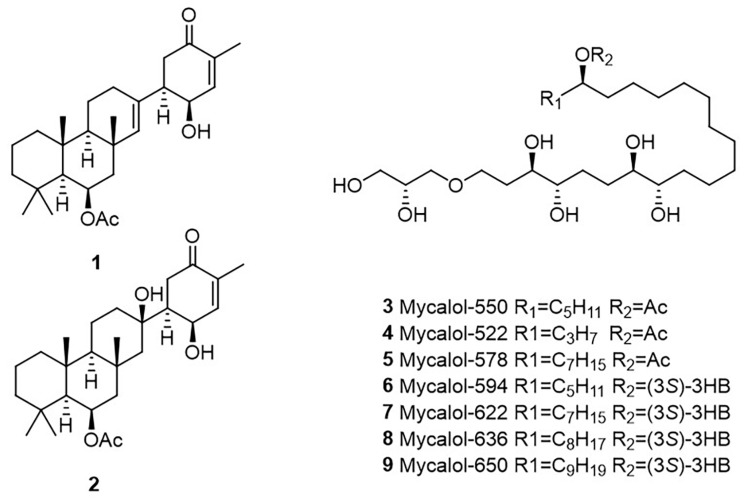
Molecular structure of suberitenones A (**1**) and B (**2**), and mycalols (**3**–**9**).

Preliminary ^1^H NMR of the active fraction D of the sponge *H. t.* (Figure S3) showed the methyl pattern that clearly indicated the presence of a sesterterpene. Further purification on silica column led to the isolation of two known compounds, suberitenone A (**1**) and B (**2**) (Figure 2), whose identification was obtained by comparing the ^1^H NMR data (Figures S4 and S5) with the literature [23], and confirmed by ESI^+^ MS analysis (Figures S6 and S7).

Chemical investigation of fraction C resulting from the SPE-fractionation of *H. d.* extract suggested the presence of polioxygenated compounds (Figure S8). Through a normal phase purification, we isolated and identified the main products family. Proton NMR spectrum (Figure S9) contained all the diagnostic signals of mycalol and its derivatives (Figure 2), previously isolated from the Antarctic sponge *Mycale (Oxymycale) acerata* [20,21,24]. HR ESI^+^-MS analysis confimed our hypothesis and allowed us to define the species composition, establishing the following percentage mycalol-522/mycalol-550/mycalo-578/mycalol-594/mycalol-622/mycalol-636/mycalol-650 as 2/54/15/3/20/2/4, respectively (Figure S10).

### 2.4. Bioactivity of Suberitenones A and B from Hemimycale topsenti and Mycalols from Haliclona (Rhizoniera) dancoi on Cancer Cell Lines

**Figure 3:**
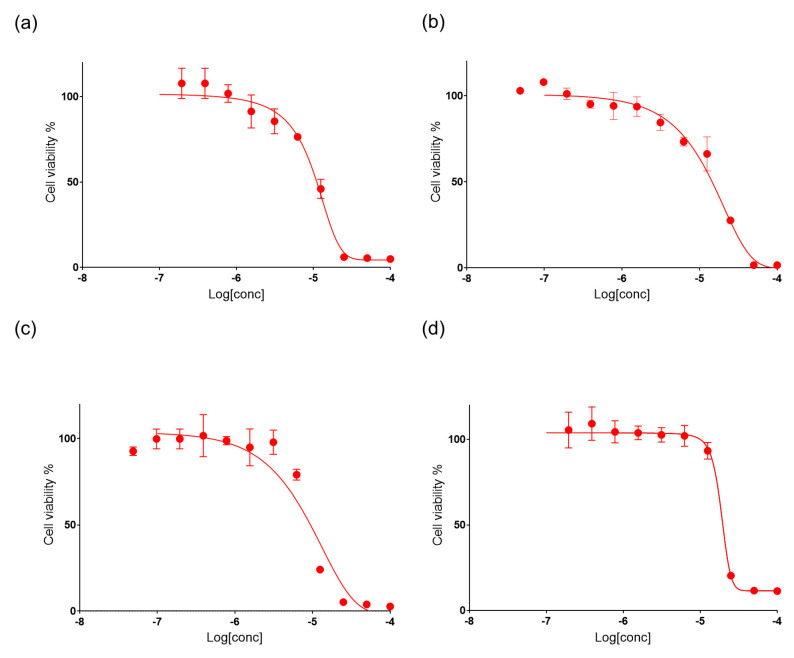
Cell viability assay. The figure shows the anti-proliferative effects of mycalols (red line) on A549 (**a**), A2058 (**b**), HepG2 (**c**), and MRC5 (**d**) cell lines, at increasing concentrations (0.05, 0.10, 0.19, 0.39, 0.78, 1.56, 3.12, 6.25, 12.5, 25, 50, 100 μM). Control cells were incubated with complete cell medium and DMSO. Results are expressed as percent survival after 72 h exposure.

The mixture of mycalols, and suberitenones A and B purified from the most active fractions were tested on cancer cell lines (A549, A2058 and HepG2) and a normal cell line (MRC5) to confirm the cytotoxic activity. In order to evaluate the IC_50_ values, the compounds were challenged at different concentrations (0.05, 0.10, 0.19, 0.39, 0.78, 1.56, 3.12, 6.25, 12.5, 25, 50, 100 μM) (Figure 3 for mycalols and Figure S11 for suberitenone A and B).

IC_50_ values of mycalols on A549, A2058, HepG2, and MRC5 cell lines were 10.1, 15.3, 9.0 and 21.3 μM, respectively (Table 2), which were roughly in line with the activity reported for the pure compounds on anaplastic thyroid carcinoma-derived FRO cells [21]. IC_50_ values of suberitenones A (**1**) on A549, A2058, HepG2, and MRC5 cells were 28.5, 10.2, 17.6, and 7.4 μM, respectively. IC_50_ values of suberitenones B (**2**) on A549, A2058, HepG2, and MRC5 were 80.7, 14.6, 19.2, and 8.5 μM, respectively (Table 2, Figure S10). The mycalols showed higher activity against cancer cell lines with respect to normal cell lines, and, in particular, they had the lowest IC_50_ when tested against HepG2 cells. On the contrary, **1** and **2** showed lower activity against cancer cell lines with respect to the normal ones (Table 2).

#### 2.4.1. Mechanism of Action

In order to elucidate the cell death metabolic pathway induced by mycalols, expression levels of selected genes by using a PCR array were analyzed in HepG2 cells treated in the presence of mycalols at 9 µM for 48 h. Gene transcription was considered affected by compounds if expression values were greater than two-fold difference with respect to the control (DMSO alone). Both differentially up-regulated and down-regulated genes are reported in Table 3. Gene expression analyses indicated that Unc-51-like kinase 1 (ULK1) and UDP-N-acetyl-alpha-d-galactosamine:polypeptide N-acetylgalactosaminyltransferase 5 (GALNT5) were the most down-regulated genes (−12.03 and −10.71-fold expression, respectively). ULK1 is involved in mammalian autophagic signaling [25] in hepatic cells and is known to be a regulator of different metabolic pathways/processes, such as mevalonate/cholesterol biosynthesis pathway [26] and lipotoxicity. Lipotoxicity is known to induce tissue damage and inflammation in metabolic disorders [27] and has been found to be related to ferroptosis [28,29], an iron-dependent programmed cell death triggered by the accumulation of lethal lipid species [30]. GALNT5 is involved in carcinogenesis and progression of choloangiocarcinoma, and activates the AKT/Erk (extracellular signal-regulated kinases) pathway [31]. In addition, a decrease in its substrate concentration (UDP-N-acetylglucosamine) has been related to ferroptosis in hepatoma cells [32]. 

In the current study, the mitogen-activated protein kinase 8 (MAPK8; also known as JNK1), which has been directly related to ferroptosis as well [33], was up-regulated (3.71-fold regulation). On the other hand, gene expression analyses showed that the pro-apoptotic genes caspase 1 (CASP1), tumor necrosis factor receptor superfamily, member 11b (TNFRSF11; known to promote apoptosis in osteoclasts, https://www.uniprot.org/uniprot/O00300) and apoptotic peptidase activating factor 1 (APAF) were down-regulated (−6.85, −5.57, and −2.03 fold regulation, respectively), while the anti-apoptotic genes nucleolar protein 3 (apoptosis repressor with CARD domain; NOL3) and B-cell lymphoma 2 A1 (BCL2A1) were the most up-regulated genes (7.40 and 5.14-fold regulation, respectively), suggesting that apoptosis was not involved in the mechanism of action of mycalol toxicity.

**Figure 4:**
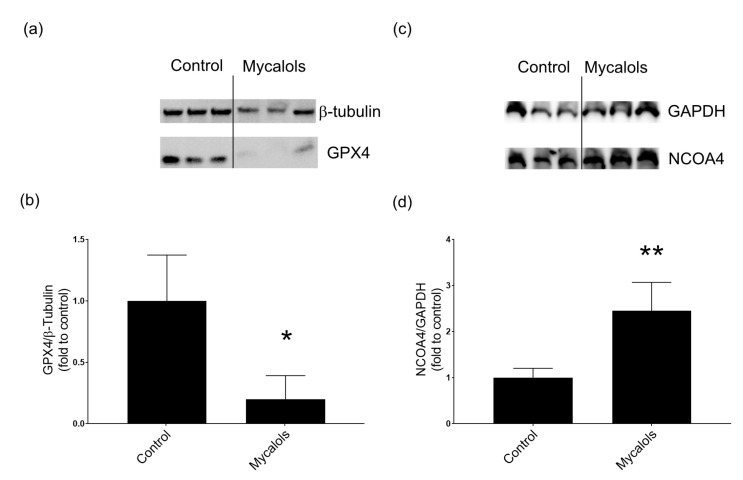
Effects of the mycalol mixture on ferroptosis-related proteins glutathione peroxidase 4 (GPX4) and nuclear receptor coactivator 4 (NCOA4). Western blotting analyses with anti-GPX4 (**a**) or anti-NCOA4 (**b**) antibody of the extracts from HepG2 treated with the DMSO alone (control) or in the presence of mycalols. The intensity of the bands of GPX4 (**c**) and NCOA4 (**d**) were normalized, respectively, with β-tubulin or glyceraldehyde 3-phosphate dehydrogenase (GAPDH), used as standard proteins, and are reported as relative to GPX4 and NCOA4 levels (* *p* < 0.05; ** *p* < 0.01).

To confirm whether mycalols triggered ferroptosis, the levels of glutathione peroxidase 4 (GPX4) and nuclear receptor coactivator 4 (NCOA4) were evaluated in hepatocellular carcinoma at the protein level. Both GPX4 and NCOA4 have an important role in ferroptosis processes. GPX4 inhibition has been associated with the accumulation of lipid peroxides, which leads to ferroptosis [29]. On the contrary, NCOA4 overexpression has been associated with an improved sensitivity to ferroptosis [34]. In the current study, the HepG2 cell line was treated in the presence or in the absence of mycalols. As shown in Figure 4a,b, the level of GPX4 protein was strongly reduced in HepG2 treated in the presence of 9 µM mycalols with respect to cells treated in the presence of the DMSO alone (*p* < 0.05). On the contrary, NCOA4 protein level was increased after mycalols treatment (*p* < 0.01; Figure 4c,d) with respect to the treatment in the presence of the vehicle alone. The reduction in GPX4 and the increase in NCOA4 protein levels suggested that mycalols were able to induce ferroptosis in hepatocarcinoma cells.

## 3. Discussion

Sponges represent an important reservoir of bioactive marine natural compounds. Abdelaleem et al. [35] reported the number of isolated bioactive compounds in a taxon of the order Demospongiae between 2013 and 2019, showing that 195 new compounds and 189 known compounds were isolated. These compounds have been reported to have a broad range of bioactivities, such as antimicrobial, cytotoxic, antioxidant, antiviral, anti-allergic, anti-parasitic, and anti-inflammatory [35].

In the current study, we have screened four Antarctic sponges belonging to the class of Demospongiae, i.e., *Mycale (Oxymycale) acerata*, *Haliclona (Rhizoniera) dancoi*, *Hemimycale topsenti*, and *Hemigellius pilosus,* for their possible cytotoxic activity against different cancer cell lines. Two of these sponges, *H. dancoi* and *H. topsenti*, have shown high cytotoxic activity. Bioactivity-guided fractionation brought to the identification of mycalols and suberitenones A and B from *H. dancoi* and *H. topsenti,* respectively.

Mycalols, polyoxygenated glyceryl alkyl ethers, have been already isolated from the Antarctic sponge *M. (Oxymycale) acerata* [20,21,24], and they have been shown to possess cytotoxic activity against thyroid cancer cells [20]. Suberitenones A and B are sesterterpenoids isolated for the first time in the Antarctic sponge *Suberites* sp. [23] and, successively, in the Antarctic sponge *Phorbas areolatus* (Thiele, 1905) [36]. Suberitenones B has shown cytotoxic activity against lung adenocarcinoma cells (A549) and hepatocellular carcinoma cells (HepG2) [36].

Here, we report a new cytotoxic activity of mycalols against A549, HepG2, and melanoma cells (A2058), other than thyroid cancer cells. In addition to A549 and HepG2, suberitenones B also showed activity against melanoma cells. Suberitenones A was shown, for the first time, to be active against lung carcinoma, hepatocellular carcinoma, and melanoma (A549, HepG2, and A2058) cells. However, both suberitenones A and B also showed high toxicity against normal cells (MRC-5, lung fibroblasts). On the contrary, as previously reported on thyroid cells [20,21], cytoxicity of mycalols was weaker against normal cells than tumor cell lines. Particularly, mycalols showed the highest activity against hepatocarcinoma cells. It is worth noting that the members of this family of products show an in vitro activity that is dependent on the length and the substitution of the alkyl skeleton, with a good correlation with the lipophilicity of the products [21]. Reasonably, this suggests that the activity of one of these products may be significantly stronger than that we recorded with the mixture. In this study, we further investigated the cell death pathway triggered by the pool of mycalols in hepatocellular carcinoma cells. Expression levels of 84 genes, involved in different death pathways, were studied. Results showed that the differentially expressed genes could be associated with ferroptosis. In particular, ULK1, GALNT5 down-regulation and MAPK8 (also known as JNK1) up-regulation have been previously related to ferroptosis processes [28,32,33,37]. Ferroptosis is a programmed cell death triggered by iron accumulation and iron-dependent lipid peroxidation. Ferroptosis has been associated to many diseases, such as cancer, kidney injury, and neurological diseases [38]. Thus, activating or blocking ferroptosis could be a strategy to treat various diseases [29,39]. GPX4 has a key role in ferroptosis and is involved in the inhibition of lipid peroxides. GPX4 converts glutathione (GSH) into its oxidized form (GSSG) and reduces lipid peroxides [29]. A marine terpenoid, named heteronemin, was recently isolated from *Hippospongia* sp., which induces apoptosis and ferroptosis in hepatocarcinoma cells, reducing GPX4 expression as well [40]. Ferroptosis has also been related to NCOA4 levels [41]. In fact, NCOA4 over-expression in cellular models increases sensitivity to ferroptosis [34]. In order to confirm that mycalols from *Haliclona (Rhizoniera) dancoi* triggered the ferroptosis pathway, we analyzed the protein levels of both GPX4 and NCOA4, in the hepatocarcinoma cell line. In cells treated in the presence of mycalols, GPX4 protein levels were significantly reduced, while NCOA4 levels increased, confirming our hypothesis. 

## 4. Conclusions

The aim of this study was to screen different sponges collected in Antarctic waters, in the framework of the XXXIV Italian National Antarctic Research Program (PNRA) in November–December 2018, in order to find bioactive natural products. Four different species of sponges have been collected and for each species, both total extracts and their four SPE-fractions have been prepared and tested on different tumor cell lines. A mixture of mycalols as well as suberitenones A and B were found in the most active fractions. Mycalols showed the highest activity. Thus, we studied the mechanism of action of the mycalol mixture at the gene and protein level, and the results suggested the activation of the ferroptosis death pathway in the hepatocarcinoma cell line. Several cancer cells have been found to modulate lipid metabolism in order to reduce ferroptosis sensitivity [42], thus targeting ferroptosis and its mediators could become a promising strategy in cancer therapy [43].

## 5. Materials and Methods

### 5.1. Sampling

Three analyzed sponge specimens were previously identified by morphological and molecular approaches in our previous paper [7]. All samples belonged to the class Demospongiae and to the following two orders: Haplosclerida with two species, *Hemigellius pilosus* (Kirkpatrick, 1907) and *Haliclona (Rhizoniera) dancoi* (Topsent, 1901), and Poecilosclerida with one species, *Mycale (Oxymycale) acerata* (Kirkpatrick, 1907). The species of sample C7 (MNA code 13860) was identified as *Hemimycale topsenti* in the present work (Table 1). Sponges were collected by scuba divers in November–December 2018 at two sites of Tethys Bay: (1) *M. (Oxymicale) acerata* at 26 m of depth (74°42.0670′ S, 164°02.5180′ E) and (2) *H. (Rhizoniera) dancoi*, *H. pilosus* and *H. topsenti* at 28 m of depth (74°40.5370′ S, 164°04.1690′ E). Samples were immediately washed with filtered and sterilized seawater to remove loosely attached bacteria and/or debris [7,44]. A small fragment of each sponge was stored in 70% ethanol for taxonomic identification, another fragment was preserved in RNA*later*^TM^ into sterile tubes and stored at −20 °C until use for DNA extraction, and the remaining sample was frozen and stored at −80 °C for chemical extraction procedures. In addition, sponge slides of spicules were deposited at the Italian National Antarctic Museum (MNA, Section of Genoa, Italy). The MNA voucher codes of each sample are reported in Table 1. 

### 5.2. Morphological Analysis and Polymerase Chain Reaction (PCR) of 18S/28S rRNA and CO1 Markers

For the taxonomic identification, small fragments of each sponge were heat-dissolved in nitric acid, rinsed in water, and dehydrated in ethanol. Then, spicules were mounted on slides for microscopic analyses, following standard methods [45]. The skeletal architecture was examined under a light microscope and hand-cut sections of sponge portions were made as described in Hooper [46]. The taxonomic classification follows the updated nomenclature reported in the World Porifera Database (WPD) [47].

About 10 mg of tissue, stored in RNA*later* at −20 °C, were excised and used for DNA extraction using the QIAamp^^®^^ DNA Micro kit (Qiagen, Hilden, Germany), according to the manufacturer’s instructions. DNA quantity (ng/μL) and quality (A260/A280; A260/A230) were evaluated by a NanoDrop spectrophotometer. PCR reactions were performed on a C1000 Touch Thermal Cycler (BioRad, Hercules, CA, USA) in a 30 µL reaction mixture by adding 1 µL of genomic DNA (starting concentration = ~100 ng/µL) from serial dilution (1:1, 1:10, 1:50 and 1:100), 6 µL of 5× Buffer GL (GeneSpin Srl, Milan, Italy), 0.6 µL of dNTPs (10 mM each), 1 µL of each forward and reverse primer (20 pmol/µL), and 0.2 µL of Xtra Taq Polymerase (5 U/µL, GeneSpin Srl, Milan, Italy). The PCR cycles were set as follows:

- for 18S and 28S, a denaturation step at 95 °C for 2 min, [35 cycles denaturation step at 95 °C for 1 min, annealing step at 52 °C (18S1/18S2; [48]), 55 °C (18S-AF/18S-BR, NL4F/NL4R; [49,50]), 57 °C (C2/D2; [51]), for 1 min and 72 °C of primer extension for 2 min], a final extension step at 72 °C for 10 min;
- CO1 primers (dgLCO1490/dgHCO2198, COX1-R1/COX1-D2; [52,53]), a first denaturation at 94 °C for 3 min, [35 cycles of denaturation at 94 °C for 30 s, annealing at 45 °C for 30 s and primer extension at 72 °C for 1 min].

PCR products were separated on 1.5% agarose gel electrophoresis in TAE buffer (40 mM Tris-acetate, 1 mM EDTA, pH 8.0) using a 100 bp DNA ladder (GeneSpin Srl, Milan, Italy) and purified using the QIAquick Gel Extraction Kit (Qiagen, Hilden, Germany) according to the manufacturer’s instructions. PCR amplicons were then sequenced in both strands through Applied Biosystems (Life Technologies, Waltham, MA, USA) 3730 Analyzer (48 capillaries). The total 18S, 28S, and CO1 regions were aligned to the nucleotide collection (GenBank, EMBL, DDBJ, PDB, RefSeq sequences) of Basic Local Alignment Search Tool (BLAST [54]) and then aligned with highly similar sequences using MultiAlin (http://multalin.toulouse.inra.fr/multalin/ accessed on June 2021) [55]).

### 5.3. Chemical Extraction and SPE Fractionation

After lyophilization, the organic material was extracted with methanol ((Merk Life Science S.r.l., Milano, Italy) at room temperature and a small amount (about 50 mg) of raw extract (hereafter referred to as TE) was subjected to SPE on a GX-271 ASPEC Gilson apparatus by using CHROMABOND^®^ HRX cartridges (6 mL/500 mg, MACHEREY-NAGEL, Düeren, Germany) as reported by Cutignano et al. [22]. Briefly, this extraction yielded five fractions (A, B, C, D, and E) eluted with H_2_O, CH_3_CN/H_2_O 7:3, CH_3_CN, and CH_2_Cl_2_/CH_3_OH 9:1, respectively. The raw extract and the SPE fractions B-E were tested on A549, A2058 and HepG2 cell lines. Fraction A mainly full of sea salt was not further analyzed. SPE fractions were monitored by TLC revealed by spraying with Ce(SO_4_)_2_.

### 5.4. Purification and Characterization of Active Compounds

#### 5.4.1. *Haliclona (Rhizoniera) dancoi (H. d.)*

The active SPE-HRX fraction C (5.1 mg) of *H. dancoi* was purified by chromatography on a silica Pasteur pipette (SiO_2_) eluted with a gradient of chloroform in methanol, to obtain 1.2 mg of mycalols mixture. ^1^H NMR in pyr-d_5_ (Figure S9) and HR ESI^+^-MS were acquired to define the chemical composition (Figure 2). 

#### 5.4.2. *Hemimycale topsenti (H. t.)*

The active SPE-HRX fraction D (2.3 mg) of *H. topsenti* was further purified by chromatography on a silica Pasteur pipette (SiO_2_) eluted with a gradient of light petroleum ether (EP) in Et_2_O (EE). Suberitenone A (**1**, 1 mg) and B (**2**, 0.4 mg) were eluted with EP/EE 8:2 and 7:3, respectively. ^1^H NMR in CDCl_3_ and HR ESI^+^-MS were acquired to define the chemical characterization (Figures S4–S7). 

### 5.5. Cell Lines

Human cells were bought at ATCC (https://www.lgcstandards-atcc.org/ accessed on June 2021). Human hepatocellular liver carcinoma (HepG2; ATCC^®^ HB-8065™) and human normal lung fibroblasts (MRC5; ATCC^®^ CCL-171™) were cultured in EMEM medium, human melanoma cells (A2058; ATCC^®^CRL-11147TM) were cultured in DMEM, adenocarcinomic human alveolar basal epithelial cells (A549; ATCC^®^CL-185TM) were cultured in F-12K medium. The media were supplemented with 10% fetal bovine serum, 50 U/mL penicillin, and 50 μg/mL streptomycin.

### 5.6. Antibody

The following antibodies were used: the rabbit monoclonal anti-glutathione peroxidase 4 (GPX4; 52455s, Cell Signaling Technology, Danvers, MA, USA), the rabbit monoclonal anti-β-tubulin (9F3, 2118s, Cell Signaling Technology, Danvers, MA, USA), the anti-rabbit IgG, HRP-linked antibody (7074s, Cell Signaling Technology, Danvers, MA, USA), the rabbit monoclonal anti-nuclear receptor coactivator 4 (NCOA4; E8H8Z, 66849s, Cell Signaling Technology, Danvers, MA, USA), the rabbit monoclonal anti-glyceraldehyde 3-phosphate dehydrogenase (GAPDH; 14C10, 2118s, Cell signaling Technology, Danvers, MA, USA), and the anti-mouse IgG, HRP-linked antibody (7074s, Cell Signaling Technology, Danvers, MA, USA).

### 5.7. In Vitro Cell Viability Studies

To evaluate the in vitro effects of the extracts or fractions on the cell viability of HepG2, A2058, A549, and MRC5 cell lines were seeded in 96-well microtiter plates at a density of 1 × 10^4^ cells/well and incubated at 37 °C to allow for cell adhesion in the plates. After 16 h, the medium was replaced with fresh medium containing increasing concentrations of extracts, fractions (0.01, 0.1, 1, 10, and 100 μg/mL) or purified compounds (0.05, 0.10, 0.19, 0.39, 0.78, 1.56, 3.12, 6.25, 12.5, 25, 50, 100 μM) dissolved in dimethyl sulfoxide (DMSO), for 72 h. The maximum concentration of DMSO used was 1% (*v/v*). Each concentration was tested at least in triplicate. After 72 h, the 3-(4,5-dimethyl-2-thizolyl)-2,5-diphenyl-2H-tetrazolium bromide (MTT; A2231,0001, AppliChem Panreac Tischkalender, GmbH, Darmstadt, Germany) assay was carried out. Briefly, the medium was replaced with a medium containing MTT at 0.5 mg/mL and the plates were incubated for 3 h at 37 °C. After incubation, the cells were treated with isopropyl alcohol (used as MTT solvent) for 30 min at room temperature. Absorbance was measured at OD = 570 nm by a microplate reader (Multiskan™ FC Microplate Photometer, Thermo Fisher Scientific, Waltham, MA, USA). Cell survival was expressed as a percentage of viable cells in the presence of the tested samples, with respect to untreated control cultures with only DMSO.

### 5.8. RNA Extraction and Reverse Transcription-Quantitative Polymerase Chain Reaction (RT-qPCR)

HepG2 cells to be used for RNA extraction were seeded in 6 wells plates (at 500,000 per well) and kept 16 h for attachment. The seeded cells were then treated in the presence of mycalols at 9 µM (molarity was evaluated based on the average molecular weights of the mycalols into the mixture) for 48 h at 37 °C. Cells were washed by adding phosphate-buffered saline (PBS 1×). 

Cells were lysed by adding 1 mL of Trisure Reagent (Meridian bioscience, Memphis, TN, USA). RNA was isolated as previously described [56]. RNA concentration, quality, and purity were assessed using an ND-1000 UV-Vis spectrophotometer (NanoDrop Technologies, Thermo Fisher Scientific, Waltham, MA, USA), monitoring the absorbance at 260 nm, and the 260/280 nm and 260/230 nm ratios (both ratios were about 2.0). RNA quality was evaluated by gel electrophoresis that showed intact RNA, with sharp ribosomal bands. About 500 ng of RNA was subjected to reverse transcription reaction using the RT2 first strand kit (cat.330401, Qiagen, Hilden, Germany) according to the manufacturer’s instructions. The RT-qPCR analysis was performed in duplicate using the RT2 Profiler PCR Array kit (cat. PAHS-212ZE-4, Qiagen, Hilden, Germany) to analyze the expression of 84 genes involved in cell death signaling pathways. Plates were run on a ViiA7 (Applied Biosystems, Foster City, CA, USA, 384-well blocks). The PCR program consisted of a denaturation step at 95 °C for 20 s followed by 40 cycles at 95 °C for 15 s and 60 °C for 1 min. The cycle threshold (Ct)-values were analyzed with PCR array data analysis online software (GeneGlobe Data Analysis Center http://pcrdataanalysis.sabiosciences.com/pcr/arrayanalysis.php accessed on May 2021, Qiagen, Hilden, Germany). Real-time data were expressed as the fold of expression, describing the changes in gene expression between cells treated in the presence of mycalols and cells treated in the presence of DMSO alone. Only expression values greater than a two-fold difference with respect to the controls were considered significant. 

### 5.9. Protein Extraction and Western Blotting Analyses

The HepG2 cells to be used for protein extraction were seeded in 6-well plates (at 500,000 per well) and kept 16 h for attachment. The seeded cells were then treated in the presence of mycalols at 9 µM for 48 h at 37 °C. Cells were washed by adding phosphate-buffered saline (PBS 1×). Cells were lysed in RIPA Buffer (Cell signaling technology, Danvers, MA, USA) 1% Triton X-100, supplemented with protease inhibitors (#310A7779, AppliChem GmbH, Darmstadt, Germany). The lysates were centrifuged at 14,000 rpm for 30 min at 4 °C. The total amount of proteins in each lysate was measured using the Bradford assay. Before sodium dodecyl sulfate-polyacrylamide gel electrophoresis (SDS-PAGE), each lysate was diluted in Laemmli sample buffer (#161-0747, Biorad, Hercules, CA, USA) containing β-mercapto-ethanol and then boiled for 5 min at 95 °C. For Western blotting analysis, proteins were transferred onto polyvinylidene difluoride (PVDF) membranes (#1704156, Trans-blot Turbo transfer pack-Biorad) using the Trans blot turbo transfer system (Biorad, Hercules, CA, USA). After the transfer, PVDF membranes were incubated in a blocking solution (1× Tris-buffered saline, TBS, 5% BSA) for 1 h at 25 °C and then incubated with primary and secondary antibodies supplemented with 5% BSA. ECL (#170-5060, Biorad, Hercules, CA, USA) reactions were performed as per the manufacturer’s instructions and immunoreactive bands were detected by chemiluminescence using ChemiDoc MP imaging system (Biorad, Hercules, CA, USA). The obtained immunoreactive bands were quantitated using Image Lab v6.0 (Biorad, Hercules, CA, USA). Different housekeeping proteins were used to normalize immunoreactive bands depending on the molecular weight of the detected proteins. Β-tubulin and GAPDH were used to normalize GPX4 and NCOA4, respectively.

## Figures and Tables

**Table 1:** Collected species and relative sample IDs and MNA codes.

Species Name	Abbreviation	Sample IDs	MNA Code
*Hemigellius pilosus* (Kirkpatrick, 1907)	*H. p.*	D4	13266
*Haliclona* (*Rhizoniera*) *dancoi* (Topsent, 1901)	*H. d.*	C6	13265
*Mycale* (*Oxymycale*) *acerata* (Kirkpatrick, 1907)	*M. a.*	B4	13264
*Hemimycale topsenti* (ex *Suberites topsenti*)	*H. t.*	C7	13860

**Table 2:** IC_50_ values of mycalols, suberitenone A and B bioactivity against A549, A2058, HepG2, and MRC5 cell lines. Values are expressed in μM.

Compounds	A549	A2058	HepG2	MRC5
Mycalols	10.1	15.3	9.0	21.3
Suberitenones A (**1**)	28.5	10.2	17.6	7.4
Suberitenones B (**2**)	80.7	14.6	19.2	8.5

**Table 3:** Transcriptional modulation of a subset of genes involved in human cell death signaling pathways in mycalols-treated HepG2 cells. Gene transcription is considered unaffected by compound treatment if fold regulation is in the range ± 2.0.

Unigene	RefSeq	Symbol	Description	Fold	SD
**Genes down-regulated by mycalols treatment**					
Hs.47061	NM_003565	ULK1	Unc-51-like kinase 1 (*C. elegans*)	−12.03	0.025
Hs.269027	NM_014568	GALNT5	UDP-*N*-acetyl-alpha-d-galactosamine:polypeptide *N*-acetylgalactosaminyltransferase 5 (GalNAc-T5)	−10.71	0.001
Hs.2490	NM_033292	CASP1	Caspase 1, apoptosis-related cysteine peptidase (interleukin 1, beta, convertase)	−6.85	0.005
Hs.484111	NM_002546	TNFRSF11	Tumor necrosis factor receptor superfamily, member 11b	−5.57	0.001
Hs.81791	NM_014592	KCNIP1	Kv channel interacting protein 1	−2.57	0.001
Hs.160562	NM_000618	IGF1	Insulin-like growth factor 1 (somatomedin C)	−2.41	0.002
Hs.552567	NM_001160	APAF1	Apoptotic peptidase activating factor 1	−2.03	0.026
**Genes up-regulated by mycalols treatment**					
Hs.513667	NM_003946	NOL3	Nucleolar protein 3 (apoptosis repressor with CARD domain)	7.40	0.029
Hs.227817	NM_004049	BCL2A1	BCL2-related protein A1	5.14	0.002
Hs.587290	NM_003900	SQSTM1	Sequestosome 1	4.75	1.256
Hs.442337	NM_176823	S100A7A	S100 calcium binding protein A7A	4.15	0.001
Hs.553833	NM_001004467	OR10J3	Olfactory receptor, family 10, subfamily J, member 3	4.05	0.001
Hs.202676	NM_014258	SYCP2	Synaptonemal complex protein 2	3.74	0.001
Hs.138211	NM_002750	MAPK8	Mitogen-activated protein kinase 8	3.71	0.132
Hs.519680	NM_001145805	IRGM	Immunity-related GTPase family, M	3.19	0.001
Hs.643440	NM_002361	MAG	Myelin associated glycoprotein	3.17	0.006
Hs.181301	NM_004079	CTSS	Cathepsin S	2.97	0.004
Hs.32949	NM_005218	DEFB1	Defensin, beta 1	2.74	0.005
Hs.29169	NM_024610	HSPBAP1	HSPB (heat shock 27kDa) associated protein 1	2.28	0.03

## Supplementary Materials

The following are available online at https://www.mdpi.com/article/10.3390/md19080459/s1, Figure S1: MultiAlin output of CO1 PCR product indicated as C7_dgLCO1490/dgHCO21 aligned to the BLAST highly similar sequence of the strain *Hemimycale topsenti* (ex *Suberites topsenti;* LN850246.1); Figure S2: Cell viability assay. The figure shows the effects on cell viability of fractions (B green bars; C blue bars; D yellow bars; E red bars) or total extracts (ex, black bars) of the samples (a) *M. a.*, (b) *H. d*., (c) *H.t*. and (d) *H. p.* on A2058, A549, and HepG2 cell lines at increasing concentrations ( 10 and 100 ng/mL and 1, 10, and 100 µg/mL). Cell viability was normalized using cells with only DMSO as a control sample. Results are expressed as percent survival after 72 h exposure (n = 3; * for *p* < 0.05; ** for *p* < 0.01 and *** for *p* < 0.001, Student’s *t*-test); Figure S3: ^1^H NMR spectrum of fraction D of the sponge *H. t*. (600 MHz, CDCl3); Figure S4: ^1^H NMR spectrum of suberitenone A **1** (600 MHz, CDCl3); Figure S5: ^1^H NMR spectrum of suberitenone B **2** (600 MHz, CDCl3); Figure S6: ESI^+^-MS spectrum of suberitenone A **1** (*m/z* [M+Na]^+^ 451.29); Figure S7: ESI^+^-MS spectrum of suberitenone B **2** (*m/z* [M+Na]^+^ 469.30); Figure S8: ^1^H NMR spectrum of fraction C of the sponge *H. d*. (600 MHz, CD_3_OD); Figure S9: ^1^H NMR spectrum of purified fraction containing mycalols (600 MHz, C_6_D_5_N); Figure S10: ESI+-MS of fraction containing mycalols (**3**–**9**); Figure S11: Cell viability antiproliferative effects induced by suberitenones A and suberitenone B. The figure shows the anti-proliferative effects of (a) suberitenones A (**1**, green line) and (b) suberitenones B (**2**, blue line) on the cell viability of A549, A2058, HepG2, and MRC5 cell lines, at increasing concentrations (0.05, 0.10, 0.19, 0.39, 0.78, 1.56, 3.12, 6.25, 12.5, 25, 50, 100 μM). The control sample contained only DMSO. Results are expressed as percent survival after 72 h exposure.
