# Supplementary material for: Bioactivity Screening of Antarctic Sponges Reveals Anticancer Activity and Potential Cell Death via Ferroptosis by Mycalols
Source: Mar Drugs. 2021 Aug 14;19(8):459. doi: 10.3390/md19080459 (PMC8400861; doi:10.3390/md19080459)

Figure S1. MultiAlin output of CO1 PCR product indicated as C7_dgLCO1490/dgHCO21 aligned to the BLAST highly similar sequence of the strain *Hemimycale topsenti* (Burton, 1929) (LN850246.1).

```
                      1        10        20        30        40        50        60        70        80        90        100       110       120       130
                      |--------+---------+---------+---------+---------+---------+---------+---------+---------+---------+---------+---------+---------|
          LN850246.1                                            GTATGTTAATTCGGTTGGAATTATCTGCGCCAGGGTCAATGTTAGGGGATGATCATTTATATAATGTTATTGTAACTGCGCATGCGTT
C7_dgLCO1490/dgHCO21  CTTTTTTTGGAGCTTTTGCTGGGANGATAGGCACTGCTTTTAGTATGTTAATTCGGTTGGAATTATCTGCGCCAGGGTCAATGTTAGGGGATGATCATTTATATAATGTTATTGTAACTGCGCATGCGTT
           Consensus  ..........................................GTATGTTAATTCGGTTGGAATTATCTGCGCCAGGGTCAATGTTAGGGGATGATCATTTATATAATGTTATTGTAACTGCGCATGCGTT

                      131      140       150       160       170       180       190       200       210       220       230       240       250       260
                      |--------+---------+---------+---------+---------+---------+---------+---------+---------+---------+---------+---------+---------|
          LN850246.1  TGTTATGATTTTTTTTTTTAGTCATGCCGGTAATGATTGGGGGATTTGGGAATTGGCTTGTGCCCTTATATATTGGGGCCCCGGATATGGCTTTTCCTCGATTAAATAATATAAGTTTTTGATTATTGCCC
C7_dgLCO1490/dgHCO21  TGTTATGATTTTTTTTTTTAGTCATGCCGGTAATGATTGGGGGATTTGGGAATTGGCTTGTGCCCTTATATATTGGGGCCCCGGATATGGCTTTTCCTCGATTAAATAATATAAGTTTTTGATTATTGCCC
           Consensus  TGTTATGATTTTTTTTTTTAGTCATGCCGGTAATGATTGGGGGATTTGGGAATTGGCTTGTGCCCTTATATATTGGGGCCCCGGATATGGCTTTTCCTCGATTAAATAATATAAGTTTTTGATTATTGCCC

                      261      270       280       290       300       310       320       330       340       350       360       370       380       390
                      |--------+---------+---------+---------+---------+---------+---------+---------+---------+---------+---------+---------+---------|
          LN850246.1  CCGGCTTTAAGTTTATTATTGGCTTCTGCTTTTGTGGAGCAAGGGGCGGGAACAGGATGGACAGTTTATCCTCCCCTATCTGGTATTCAGGCCCACTCAGGCGGTTCAGTCGATTTAGTAATATTTAGTT
C7_dgLCO1490/dgHCO21  CCGGCTTTAAGTTTATTATTGGCTTCTGCTTTTGTGGAGCAAGGGGCGGGAACAGGATGGACAGTTTATCCTCCCCTATCTGGTATTCAGGCCCACTCAGGCGGTTCAGTCGATTTAGTAATATTTAGTT
           Consensus  CCGGCTTTAAGTTTATTATTGGCTTCTGCTTTTGTGGAGCAAGGGGCGGGAACAGGATGGACAGTTTATCCTCCCCTATCTGGTATTCAGGCCCACTCAGGCGGTTCAGTCGATTTAGTAATATTTAGTT

                      391      400       410       420       430       440       450       460       470       480       490       500       510       520
                      |--------+---------+---------+---------+---------+---------+---------+---------+---------+---------+---------+---------+---------|
          LN850246.1  TACATTTAGCGGGAATTTCTTCTATTTTAGGTGCTATGAACTTTATTACTACAATAATAAACATGCGGGCATTGGGGGTAACTTTGGATAGAATGCCTTTATTCGTTTGATCGATTTTAGTTACTGCTAT
C7_dgLCO1490/dgHCO21  TACATTTAGCGGGAATTTCTTCTATTTTAGGTGCTATGAACTTTATTACTACAATAATAAACATGCGGGCATTGGGGGTAACTTTGGATAGAATGCCTTTATTCGTTTGATCGATTTTAGTTACTGCTAT
           Consensus  TACATTTAGCGGGAATTTCTTCTATTTTAGGTGCTATGAACTTTATTACTACAATAATAAACATGCGGGCATTGGGGGTAACTTTGGATAGAATGCCTTTATTCGTTTGATCGATTTTAGTTACTGCTAT

                      521      530       540       550       560       570       580       590       600       610       620       630       640       650
                      |--------+---------+---------+---------+---------+---------+---------+---------+---------+---------+---------+---------+---------|
          LN850246.1  TTTATTATTATTATCTTTACCCGTATTAGCAGGGGCTATTACTATGTTATTAACAGATAGAAATTTTAATACTGCCTTTTTTGATCC
C7_dgLCO1490/dgHCO21  TTTATTATTATTATCTTTACCCGTATTAGCAGGGGCTATTACTATGTTATTAACAGATAGAAATTTTAATACTGCCTTTTTTGATCCTGCAGGAGGAGGGGATCCTATCTTATATCAACATTTGTTTTGA
           Consensus  TTTATTATTATTATCTTTACCCGTATTAGCAGGGGCTATTACTATGTTATTAACAGATAGAAATTTTAATACTGCCTTTTTTGATCC...........................................

                      651      660663
                      |--------+--|
          LN850246.1
C7_dgLCO1490/dgHCO21  TTNTTTGGTCACC
           Consensus  .............
```

Figure S2. Cell viability assay. The figure shows the effects on cell-viability of fractions (B green bars; C blue bars ; D yellow bars; E red bars) or total extracts (Ex, black bars) of the samples (a) M.a., (b) H. d., (c) H.t. and (d) H. p. on A2058, A549 and HepG2 cell lines at increasing concentrations (10 and 100 ng/mL and 1, 10 and 100 μg/mL). Cell viability was normalized using cells with only DMSO as control sample. Results are expressed as percent survival after 72 h exposure (n = 3; * for p<0,05; ** for p < 0.01 and *** for p < 0.001, Student's t-test).

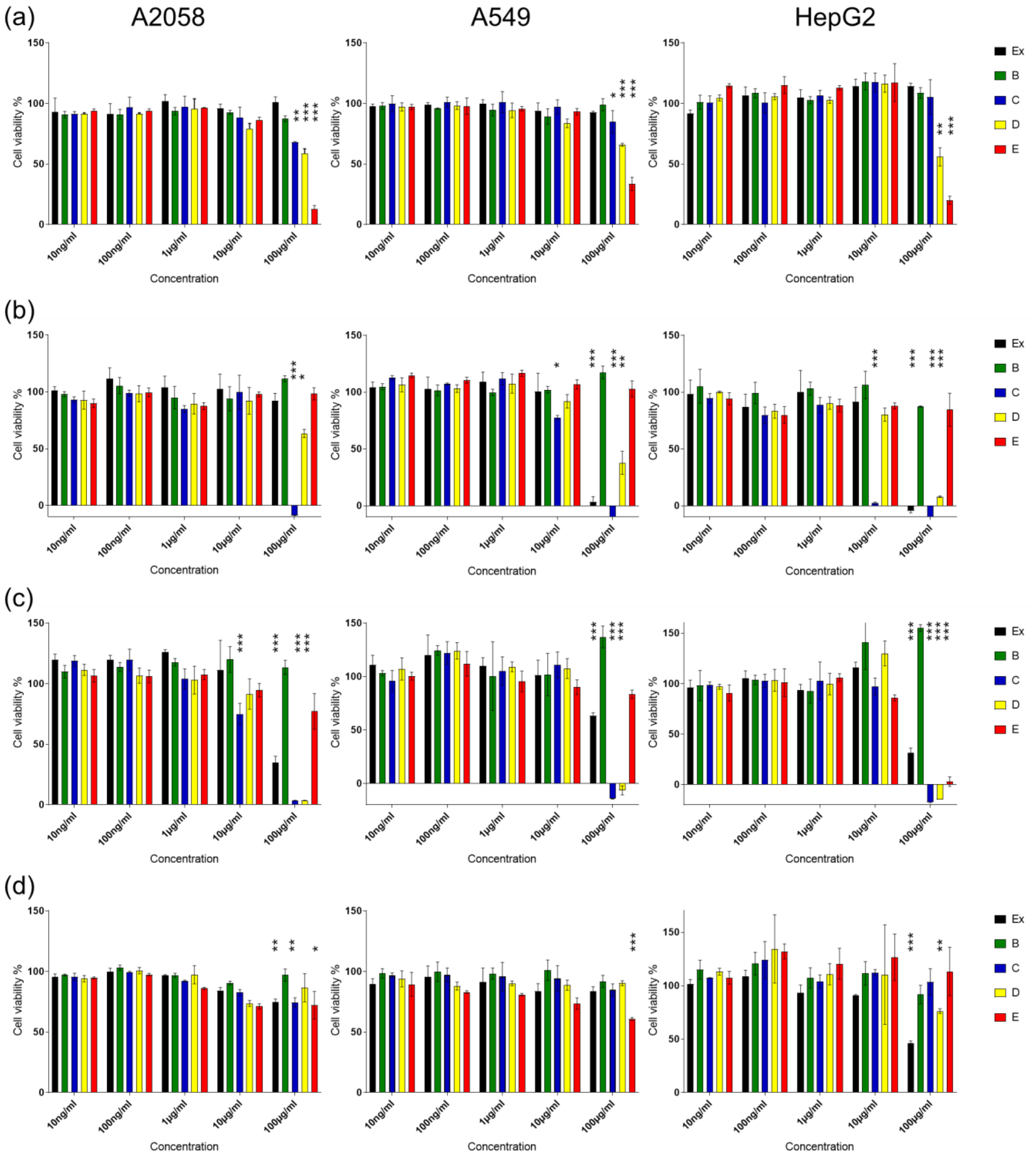

Figure S3. ^{1}H NMR spectrum of fraction D of the sponge H. t. (600 MHz, CDCl3).

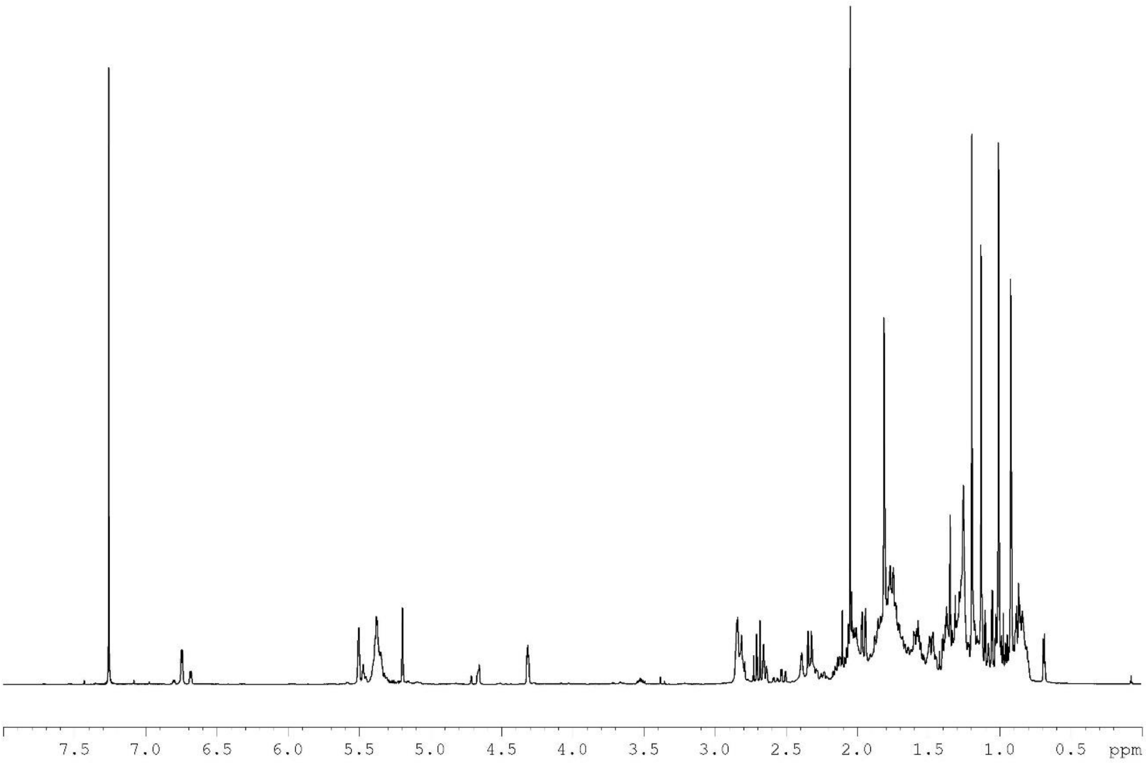

Figure S4. ^{1}H NMR spectrum of suberitenone A **1** (600 MHz, CDCl3).

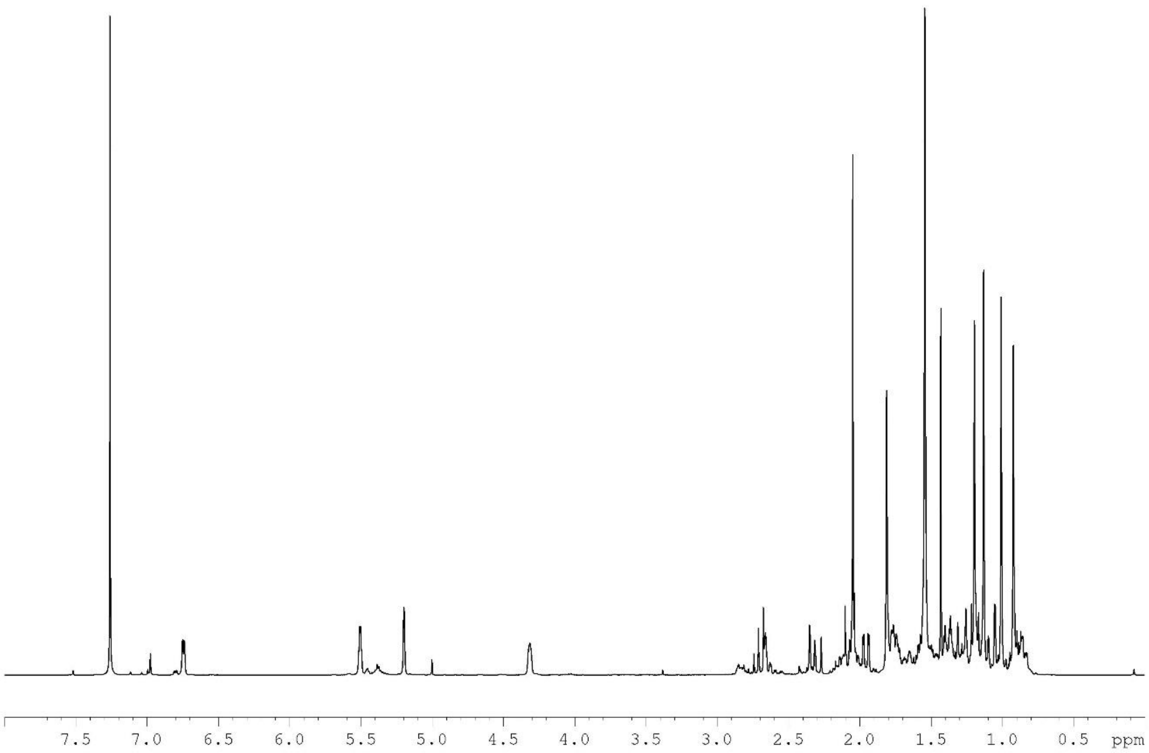

Figure S5. ^1H NMR spectrum of suberitenone B **2** (600 MHz, CDCl3).

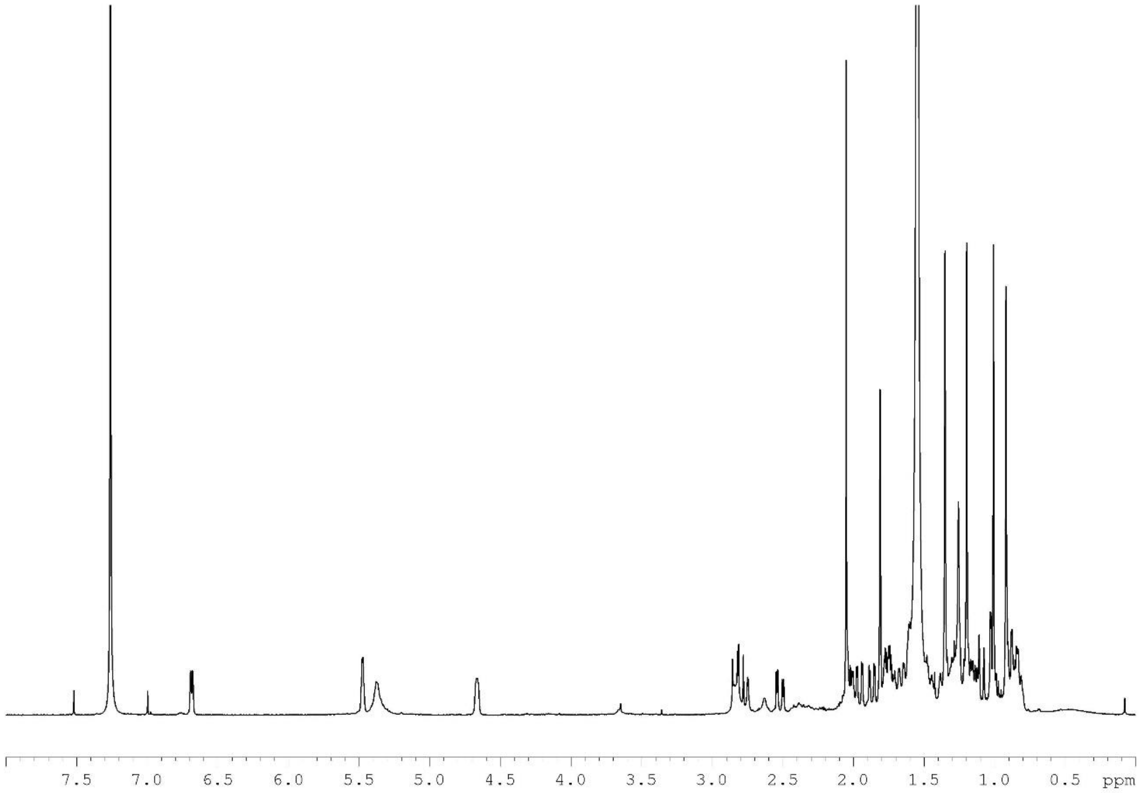

Figure S6. ESI$^+$-MS spectrum of suberitenone A **1** (m/z [M+Na]$^+$ 451.29).

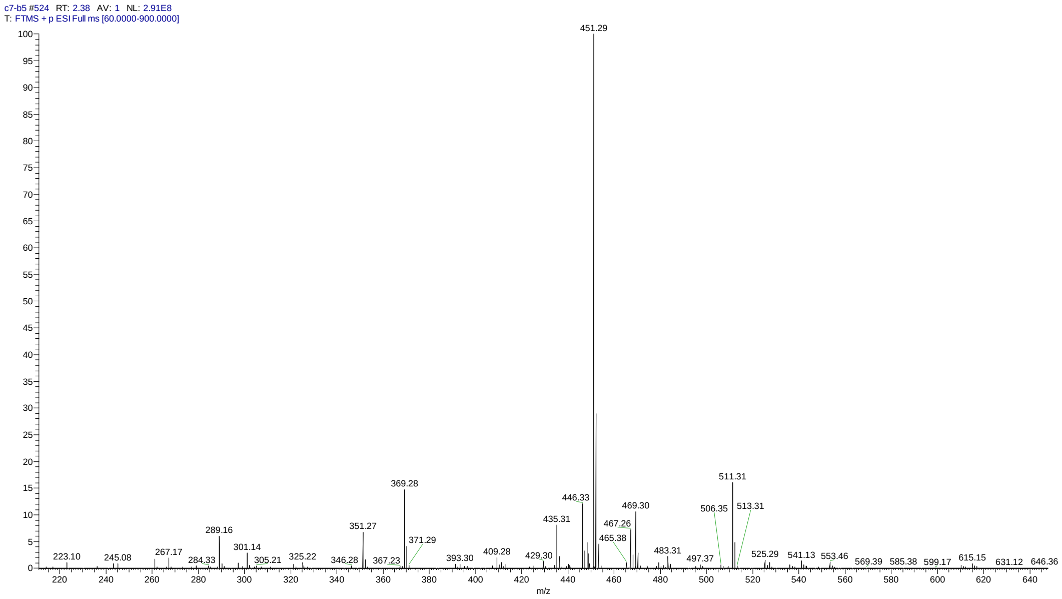

Figure S7. ESI+-MS spectrum of suberitenone B **2** (*m*/*z* [M+Na]+ 469.30).

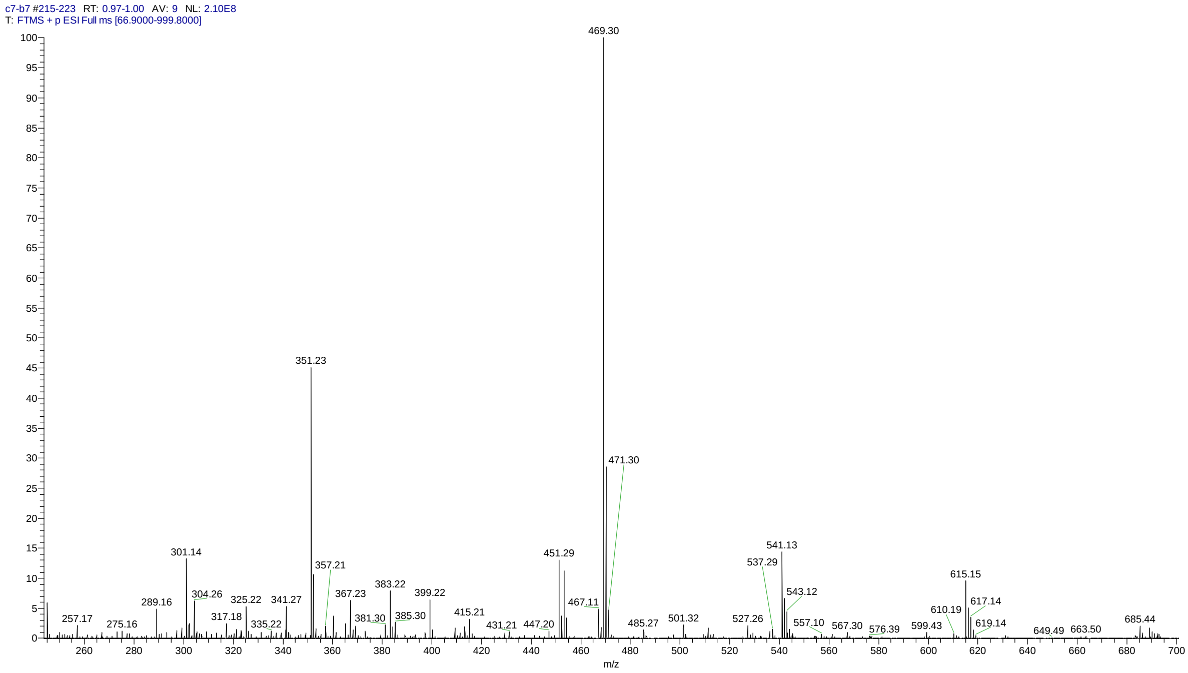

Figure S8. ^{1}H NMR spectrum of fraction C of the sponge H. d. (600 MHz, CD_3OD).

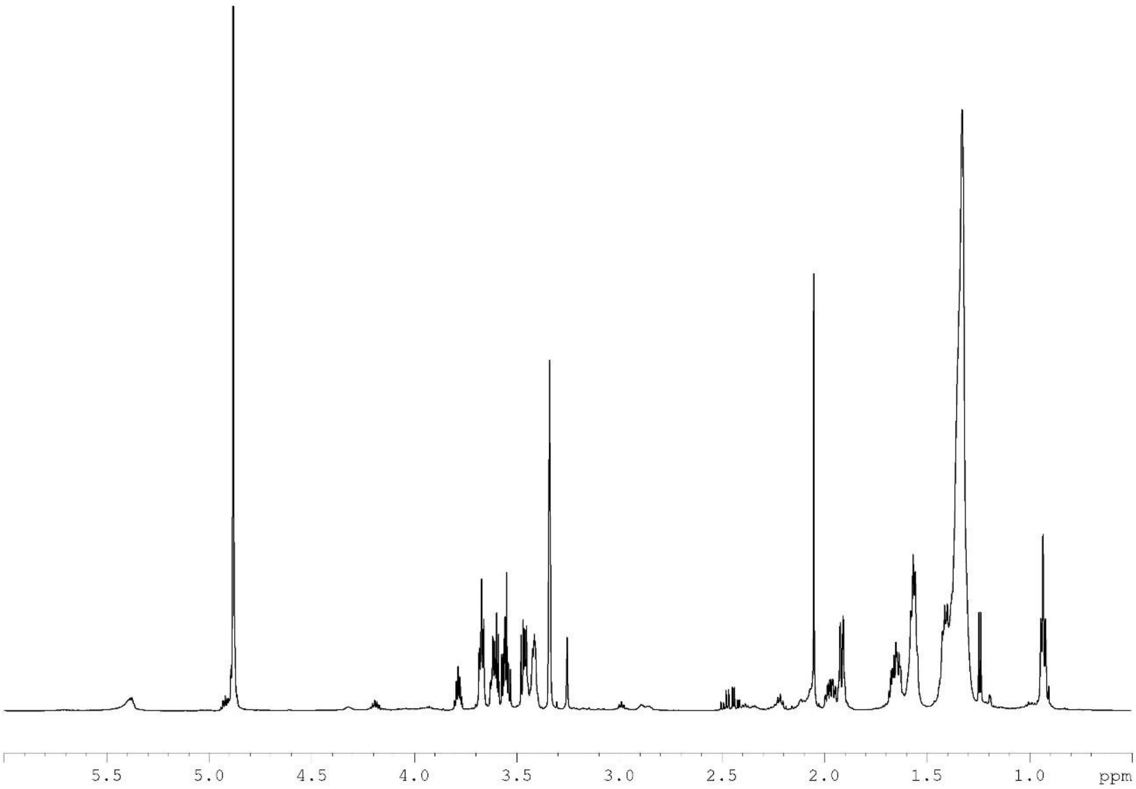

Figure S9. ^{1}H NMR spectrum of purified fraction containing mycalols (600 MHz, C_6D_5N).

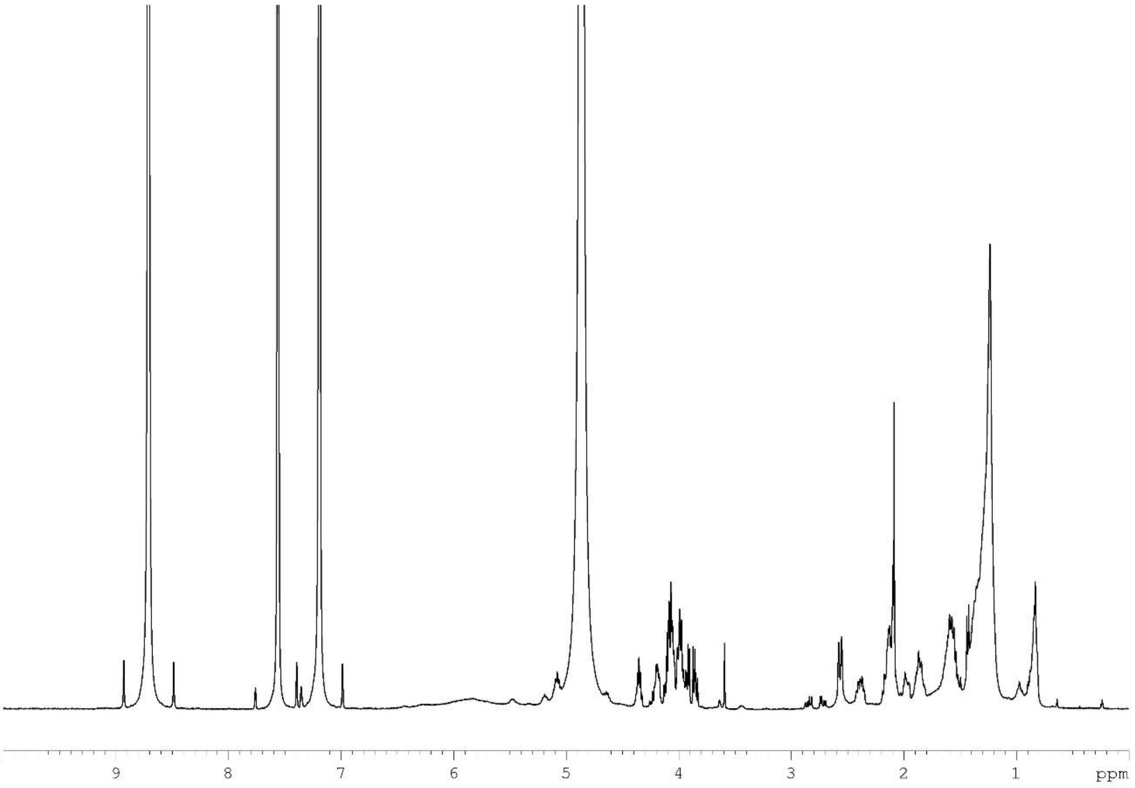

Figure S10. ESI^{+}-MS of fraction containing mycalols (**3-9**).

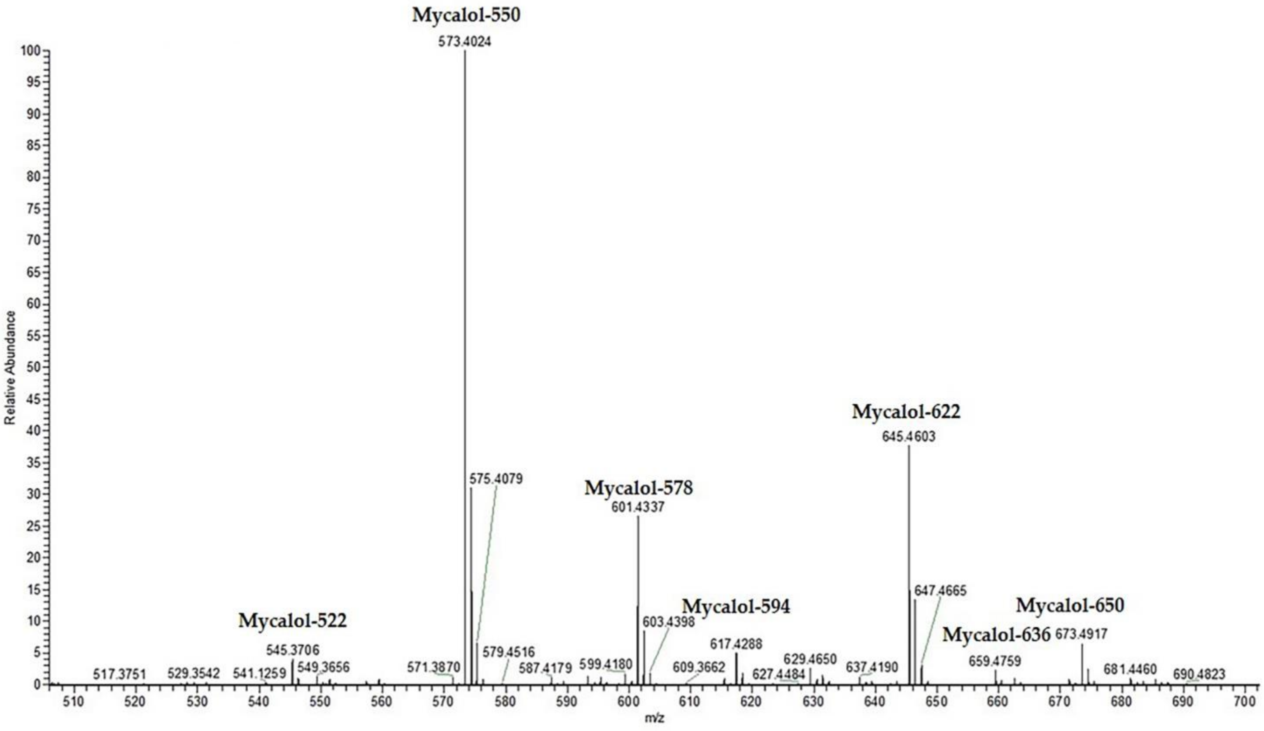

Figure S11. Cell viability effects induced by suberitenones A and suberitenone B. The figure shows the effects of (a) suberitenones A (**1**, green line) and (b) suberitenones B (**2**, blue line) on cell viability of A549, A2058, HepG2 and MRC5 cell lines, at increasing concentrations (0.05, 0.10, 0.19, 0.39, 0.78, 1.56, 3.12, 6.25, 12.5, 25, 50, 100 μM). Control sample contained only DMSO. Results are expressed as percent survival after 72 h exposure**.**

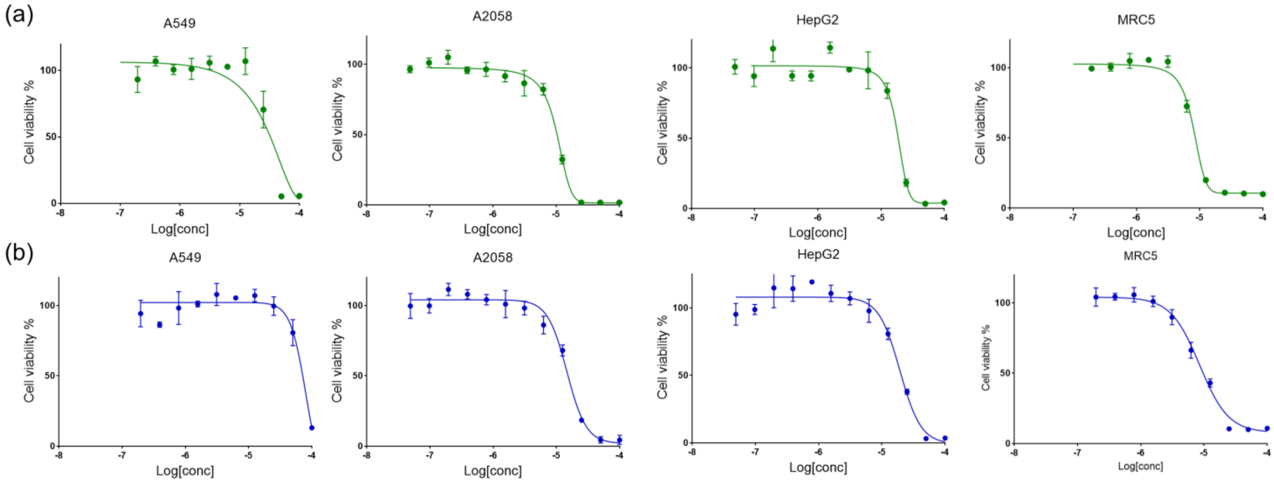

Supplement: Supplementary file 1 [file marinedrugs-19-00459-s001.zip › marinedrugs-1318122-supplementary.pdf]
